# Microbial diversity of saline environments: searching for cytotoxic activities

**DOI:** 10.1186/s13568-017-0527-6

**Published:** 2017-12-22

**Authors:** Carolina Díaz-Cárdenas, Angela Cantillo, Laura Yinneth Rojas, Tito Sandoval, Susana Fiorentino, Jorge Robles, Freddy A. Ramos, María Mercedes Zambrano, Sandra Baena

**Affiliations:** 1 Unidad de Saneamiento y Biotecnología Ambiental, Departamento de Biología, Pontificia Universidad Javeriana, POB 56710, Bogotá DC, Colombia; 2 Corporación Corpogen, Carrera 5 # 66A-34, Bogotá DC, Colombia; 3 Grupo de Inmunobiología y Unidad de Investigación en Ciencias Biomédicas, Pontificia Universidad Javeriana, POB 56710, Bogotá DC, Colombia; 4 Grupo de Investigación Fitoquímica, Pontificia Universidad Javeriana, POB 56710, Bogotá DC, Colombia; 5 Departamento de Química, Universidad Nacional de Colombia-Sede Bogotá, Carrera 30 # 45-03, Bogotá DC, Colombia

**Keywords:** Phylogenetic diversity, Halophilic bacteria, Cytotoxic activity, Secondary metabolism

## Abstract

In order to select halophilic microorganisms as a source of compounds with cytotoxic activities, a total of 135 bacterial strains were isolated from water and sediment samples collected from the Zipaquirá salt mine in the Colombian Andes. We determined the cytotoxic effects of 100 crude extracts from 54 selected organisms on the adherent murine mammary cell carcinoma 4T1 and human mammary adenocarcinoma MCF-7 cell lines. These extracts were obtained from strains of *Isoptericola, Ornithinimicrobium, Janibacter, Nesterenkonia, Alkalibacterium, Bacillus, Halomonas, Chromohalobacter, Shewanella, Salipiger, Martellela, Oceanibaculum, Caenispirillum* and *Labrenzia*. The extracts of 23 strains showed an IC_50_ of less than 100 μg mL^−1^. They were subsequently analyzed by LC/MS allowing dereplication of 20 compounds. The cytotoxic effect was related to a complex mixture of diketopiperazines present in many of the extracts analyzed. The greatest cytotoxic activity against both of the evaluated cell lines was obtained from the chloroform extract of *Labrenzia aggregata* USBA 371 which had an IC_50_ < 6 μg mL^−1^. Other extracts with high levels of cytotoxic activity were obtained from *Bacillus* sp. (IC_50_ < 50 μg mL^−1^) which contained several compounds such as macrolactin L and A, 7-*O*-succinoylmacrolactin F and iturin. *Shewanella chilikensis* USBA 344 also showed high levels of cytotoxic activity against both cell lines in the crude extract: an IC_50_ < 15 μg mL^−1^ against the 4T1 cell line and an IC_50_ < 68 μg mL^−1^ against the MCF-7 cell line. *Nesterenkonia sandarakina* CG 35, which has an IC_50_ of 118 µg mL^−1^ against 4T1, is a producer of diketopiperazines and 1-acetyl-β-carboline. Also, *Ornithinimicrobium kibberense* CG 24, which has IC_50_ < 50 μg mL^−1^, was a producer of diketopiperazines and lagunamycin. Our study demonstrates that these saline environments are habitats of halophilic and halotolerant bacteria that have previously unreported cytotoxic activity.

**Electronic supplementary material:**

The online version of this article (10.1186/s13568-017-0527-6) contains supplementary material, which is available to authorized users.

## Introduction

Saline environments are usually defined as those containing salt concentrations similar to seawater (~ 3.5% (w/v) total dissolved salts) whereas hypersaline environments contain higher salt concentrations and these environments are largely used for the study of the microbial diversity and ecology (Ventosa et al. 2014). Halophiles have attracted the interests of researchers because of their adaptability to a wide range of salinities as well as their potentially promising applications. They are sources of compatible solutes, stable enzymes (DNAses, lipases, amylases and proteases), bacteriorhodopsin, polymers, β-carotene and other organic substances of interest (Chen et al. 2010; Oren 2010; Braña et al. 2015; da Silva et al. 2015).

These microorganisms have been also considered as a potential source of bioactive compounds. Several antitumor and antimicrobial substances have already been isolated from moderately and extremely halophilic microorganisms including archaeal proteinaceous antimicrobials (i.e. halocins) that have been isolated from several extremely halophilic archaea such as *Natrinema* sp. (Karthikeyan et al. 2013) and *Haloferax mediterranei* (O’Connor and Shand 2002). Other examples are pigments such as prodigiosin isolated from *Vibrio* spp., that exhibit antimicrobial activity (Gallardo et al. 2016). Lipopeptides, polyketides, terpenes, macrolactins from diverse microorganisms such as *Saccharothrix* sp., *Nocardiopsis* sp., and *Bacillus* spp. (Gan et al. 2015; Son et al. 2016; Kim et al. 2017) and diketopiperazines (DKPs) from *Streptomyces* spp., *Bacillus* spp. and *Nocardiopsis* spp. (Raju et al. 2009; Fu et al. 2011; Yonezawa et al. 2011; Gu et al. 2013). Thus, the saline environments could be largely underexplored ecological niches for the discovery of bioactive metabolites and these halophilic microorganisms are potential sources for a broad range of new therapeutic compounds (Demain 2014). The current estimate that 90% of the biosynthetic capacity of microorganisms is yet to be discovered highlights the importance of research in microbial diversity and in the discovery of bioactive principles as keys to unlocking the metabolic potential of microbes (Walsh and Fischbach 2010).

The aim of this study was to generate information on the cytotoxic potential of extracts and compounds produced by halophilic and halotolerant organisms isolated from unexplored Colombian rock salt and saline spring environments. Thus, in this study, halophilic microorganisms were isolated from hypersaline environments at the Zipaquirá salt mine, located in the eastern Andean Mountain range. They were then analyzed for their capacities to produce cytotoxic compounds under several salt concentrations.

## Materials and methods

### Zipaquirá salt mine: site description and sampling

Samples of water, sediment, brine and solid (rock salt) were collected in 500 mL sterile glass and plastic bottles from six sampling points during three sampling events at the Zipaquirá salt mine in Colombia (5°01′06.18″N and 74°0′13.63″W) at 2656 m.a.s.l. The samples were used to inoculate basal salt medium, to which several carbon sources were added. The samples were carefully stored at 4 °C and transported to the laboratories prior to analysis. The samples were used for enrichment on the following day.

For physical–chemical analysis, 5 L of water from each sampling point were collected. The temperature and pH analyses were performed in situ using a Hach pH meter. The analysis of calcium (Ca^2+^ mg L^−1^), total organic carbon (mg L^−1^), chlorides (Cl^−^ mg L^−1^), total phosphorous (PO_4_^3−^ mg L^−1^), total iron (Fe mg L^−1^), magnesium (Mg^2+^ mg L^−1^), manganese (Mn^2+^ mg L^−1^), nitrates (N-NO_3_ mg L^−1^), ammoniacal nitrogen (N-NH_4_ mg L^−1^), potassium (K^+^ mg L^−1^), salinity (conductivity mS cm^−1^), sodium (Na^+^ mg L^−1^), sulfate (SO_4_^2−^ mg L^−1^) and sulfites (SO_3_^2−^ mg L^−1^) was performed using standard methods (APHA/AWWA/WEF 2012).

### Strains

Both halophilic microorganisms isolated from Zipaquirá salt mine and other previously isolated strains were used for screening in this study. These previously isolated strains were obtained in a previous study from two saline springs in the Central Mountain Range of the Colombian Andes and are part of our collection of microorganisms (Díaz-Cárdenas and Baena 2015): *Oceanibaculum indicum* USBA 36, *Caenispirillum bisanense* USBA 85, *Shewanella chilikensis* USBA 344 and *Labrenzia aggregata* USBA 371. All of the strains evaluated for the cytotoxic screenings were deposited in the *Colección de microorganismos de la Pontificia Universidad Javeriana* (WDCM857).

### Isolation and enrichment of halophilic microorganisms

In order to isolate halophilic microorganisms the following media were used. (1) Actinomycete isolation agar (per liter of distilled water): 4% (w/v) NaCl, 2.0 g of sodium caseinate, 0.1 g of asparagine, 4.0 g of sodium propionate, 0.5 g of K_2_HPO_4_, 0.1 g of MgSO_4_·7H_2_O, 0.1 g of FeSO_4_, 5.0 g of glycerol and 15 g of agar (Sigma). (2) Halophilic medium (HM) (per liter of distilled water): 10  or 4% (w/v) NaCl, 2.0 g of KCl, 1.0 g of MgSO_4_, 0.36 g of CaCl_2_^ .^ 2H_2_O, 0.23 g of NaBr, 0.06 g of NaHCO_3_, trace FeCl_3_, 10.0 g of yeast extract (Difco), 5.0 g of peptone (Difco) and 1.0 g of glucose (pH 7.5) (Ventosa Ucero et al. 1982). (3) M63 medium (per liter of distilled water): 13.6 g of KH_2_PO_4_, 2 g of (NH_4_)_2_SO_4_, 0.5 g of FeSO_4_·7H_2_O, 0.24 g of MgSO_4_·7H_2_O and 4 g of glucose (Takashina et al. 1994). (4) Water from the sampling points filtered through 47 mm diameter filters with a pore size of 0.45 μm (GTTP, Millipore, Billerica, MA, USA) and then sterilized at 121 °C and 15 psi for 30 min. This water was supplemented with 1 ml L^−1^ of the oligoelement solution SL10 (Widdel et al. 1983) and either 0.1% (w/v) casamino acids, 10 mM glycerol or 4 mM acetic acid as the carbon source. The culture medium pH was adjusted to 6.0–7.0 with 10% NaOH (w/v) (Merck), to take into account the in situ pH at the sampling points. Enrichments were performed by inoculating 1 mL of sample from each of the six sampling points into tubes containing 5 mL of culture media and then incubating the tubes at room temperature (22 °C ± 3) in the dark and without agitation until growth was observed. Microbial growth was inspected daily for 2 weeks by light microscopy using a Nikon phase contrast microscope (Nikon i50 Nikon, Melville, NY, USA). After confirmation of growth, tenfold serial dilutions were prepared and inoculated into tubes with 5 mL of culture medium, and the last positive serial dilution was used to inoculate plates containing enrichment medium fortified with 2% (w/v) agar (Sigma). The tubes were incubated under the same conditions described above. Cells with different morphologies and appearances were transferred to new culture plates to obtain pure cultures. The isolates were purified by repeated growth on solid media and preserved with 20% (v/v) glycerol at − 80 °C.

Solid samples (salt rock) were first surface-sterilized by immersing 200 g of the sample in ethanol followed by flaming. Each 200 g sample was then introduced into a 250-mL Erlenmeyer flask with 100 mL of liquid medium supplemented with various NaCl concentrations (5, 8, 10 and 20% w/v) and then incubated in the dark without stirring for 10 days at room temperature. Serial dilutions of these 10 day cultures were then plated onto TSA (Merck) and HM halophilic medium supplemented with 5 or 8% (w/v) NaCl; 10 day cultures in 10 and 20% (w/v) NaCl were plated directly onto solid media (100 µL). After 5 days of incubation at room temperature in the dark, colonies with morphological and pigment differences were selected, and successive passages were conducted until pure strains were obtained.

The isolated strains were grown in TSB (Merck), marine broth (Difco) or modified marine broth (Difco) supplemented with either 0.1% (w/v) casamino acids or 5 mM glucose, and 3, 5 or 8% (w/v) NaCl to adjust the salinity.

#### Sequence analysis of the 16S rRNA gene and phylogenetic reconstruction

DNA was extracted using the Wizard^®^ Genomic DNA kit (Promega cat #A1120) from 10 mL of an exponentially growing microbial culture at 8000×*g* for 10 min or purified from colonies resuspended in 10 mM Tris–HCl and 0.7% SDS (Cayol et al. 1995). The 16S rRNA gene was amplified using the universal primers 27F (5′-AGAGTTTGATCMTGGCTCAG-3′) and 1492R (5′-GGTTACCTTGTTACGACTT-3´) (Lane 1991) for bacteria and 344F (5′-ACKGCTCAGTAACACGT-3′) (Raskin et al. 1994) and 915R (5′-GTGCTCCCCCGCCAATTCCT-3′) (Stahl and Amann 1991) for archaea. All PCRs (in a 50-µL total volume reaction) contained < 0.5 µg of DNA template, 0.2 μM of each primer, 0.2 mM dNTPs, 1.5 mM MgCl_2_, 1X Buffer and 1.25 U of GoTaq^®^ DNA Polymerase (Promega). The PCR conditions were 94 °C for 2 min, 35 cycles at 94 °C for 30 s, 55 °C for 45 s, and 72 °C for 1 min followed by a final elongation step of 10 min at 72 °C. Sequencing was performed using an ABI PRISM^®^ 3500 (Laboratorio de secuenciación de ADN, Universidad de Los Andes, Bogotá, Colombia) and an ABI PRISM^®^ 3730XL Analyzer (Macrogen Inc., South Korea). Raw sequence data were imported into BioEdit, version 7.2.5, sequence editor (Hall 1999) and corrected manually for errors. Sequences were compared against sequences of type strains using RDP release 11 (https://rdp.cme.msu.edu/).

#### Nucleotide sequence accession numbers

The 16S rRNA gene sequences of the novel and previously isolated strains were deposited in GenBank under the accession numbers MF197926–MF197981, MF506732–MF506815.

### Cytotoxic activity screening assays

Cultures were started with a saturated (24–120 h) culture, diluted to 10^5^ CFU and inoculated 1:100 into fresh medium, and cultured under the conditions described in Additional file 1: Table S1. The culture media used were marine broth (Difco), modified marine broth with 1% (w/v) casamino acids, 5 mM glucose, TSB (Merck) and TSB supplemented with 3% (w/v), 4% (w/v), 5% (w/v) or 8% (w/v) NaCl. All cultures were grown in duplicate.

#### Preparation of crude extracts

Microbial cultures were centrifuged at 9000×*g* at 4 °C for 20 min, and cell-free supernatants were sequentially extracted three times with chloroform and ethyl acetate at a 1:1 ratio. The extracts were evaporated under reduced pressure using a Buchi Rotavapor R114 (Buchi, Switzerland) and then stored at − 20 °C in amber glass vials prior to use. Uninoculated culture medium was processed as a negative control. The chloroform extracts were resuspended in dimethyl sulfoxide (DMSO), and the ethyl acetate extracts were resuspended in ethanol at final concentrations of 25 mg mL^−1^ (stock solution). Subsequently, the samples were diluted (from 250 to 31 μg mL^−1^) for evaluation of cytotoxic activity.

#### Cytotoxic activity screening

The cytotoxic activity of the extracts was determined using the neutral red technique (Repetto et al. 2008). Briefly, the adherent 4T1 (mouse mammary tumor) and MCF-7 (human mammary adenocarcinoma) cell lines were cultured in RPMI medium supplemented with 10% fetal bovine serum in 96-well plates at a density of 3 × 10^3^ cells well^−1^. The plates were incubated for 12 h at 37 °C in 5% CO_2_ to allow monolayer formation. Selection of promising strains was performed using four dilutions ranging from 250 to 31.25 μg mL^−1^. The solvent used in the reconstitution of the extract (ethanol or DMSO) was evaluated as a negative control for each test in the same volume as the extracts. Doxorubicin at a maximum concentration of 5 μM was used as a positive control. In addition, untreated cell controls, cell-free treatments and a culture medium control were included for each extract. These analyses were performed in duplicate.

After 48 h of treatment, two washes were performed with 1X PBS, the neutral red reagent was added to each well and the plates were incubated for 3 h. After incubation, two washes were performed with PBS. Finally, a bleach solution (1% (v/v) glacial acetic acid, 50% (v/v) ethanol and 49% (v/v) distilled water) was added to each well and stirred for 20 min before the absorbance was read at 540 nm (Multiskan™ FC microplate photometer) (Repetto et al. 2008).

Inhibitory concentration 50 (IC_50_) values were defined as the concentration of the extracts that generated 50% inhibition of tumor cell growth. To calculate the IC_50_, the same procedure described for the initial screening was performed using eight decreasing concentrations starting at 250 μg mL^−1^. The positive and negative controls were the same as those used for the initial screening. The calculation was performed using a non-linear regression method of Log (concentration) vs. percentage inhibition using algorithms with three or four parameters according to the behavior of the treatment. These analyses were conducted using GraphPad Prism 6.0.

### Fractionation and metabolomics evaluation

Crude extracts with cytotoxic activity (100 mg) were fractionated using cartridges (Hypersep DIOL—Thermo Scientific) and solvents with increasing polarity, starting with hexane and ending with methanol. Twelve fractions were obtained as follows: F1.1 and F 1.2. (100% hexane), F2.1 and F2.2 (hexane/ethyl acetate, 8:2), F3.1 and F3.2 (hexane/ethyl acetate 1:1), F4.1 and F4.2 (100% ethyl acetate), F5.1 and F5.2 (ethyl acetate/methanol, 1:1) and F6.1 and F6.2 (100% methanol). The fractions were evaporated at room temperature and stored at − 20 °C in amber glass vials prior to use.

### LC/MS analysis of active extracts and fractions

Selected samples were analyzed by liquid chromatography-mass spectrometry (LC/MS) at Fundación MEDINA in Spain as described by (González-Menéndez et al. 2016). The LC analysis was performed on an Agilent 1200 Rapid Resolution HPLC interfaced to a Bruker maXis mass spectrometer. The volume of sample injected was 2 µL. A Zorbax SB-C8 column (2.1 × 30 mm, 3.5 µm particle size) was used for separation during which the column was maintained at 40 °C with a flow rate of 300 μL min^−1^. Two solvents were used as the mobile phase. Solvent A consisted of 10% acetonitrile and 90% water and solvent B consisted of 90% acetonitrile and 10% water. Both had 13 mM ammonium formate and 0.01% (v/v) trifluoroacetic acid. The gradient started at 10% B rose to 100% B in 6 min, was maintained at 100% B for 2 min and returned to 10% B for 2 min to initialize the system. Full diode array UV scans from 100 to 900 nm were collected in 4-nm steps at 0.25 s scan^−1^. Mass spectrometry acquisition to generate raw data was performed on an Agilent MSD 1100 mass spectrometer. Ionization was achieved by electrospray ionization (ESI) in positive mode. The instrumental parameters were: 4 kV capillary voltage, drying gas flow of 11 L min^−1^ at 200 °C and nebulizer pressure at 2.8 bars. TFA-Na cluster ions were used for mass calibration of the instrument prior to sample injection. Each sample run was recalibrated by infusion with the same TFA-NA calibrant before the chromatographic front. Database matching was performed using an in-house developed application within which the UV signal, retention time, mass signal and molecular formula of the selected ions were compared to the UV-HPLC-HRMS data of known metabolites stored in the Fundación MEDINA database (High Resolution MS 647 of Actinobacteria, 384 of Fungi and 42 of Plants) and matching molecular formulas in the Chapman and Hall Dictionary of Natural Products database (290,000 entries of natural products) (Running 1993).

## Results

### Isolation of halophilic microorganisms from Zipaquirá salt mine and phylogenic diversity of isolated bacteria

**Fig. 1:**
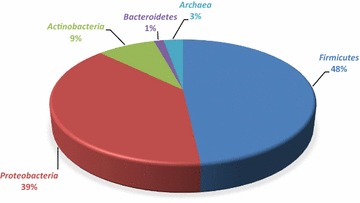
Distribution of the relative abundance of the *Bacteria* and *Archaea* strains isolated at Zipaquirá salt mine

In order to isolate halophilic microorganisms, we collected water, sediment, brine and solid (rock salt) samples from six sites in a salt mine in Zipaquirá, Colombia. This environment developed as a salt dome around 250 million years ago and then was raised above sea level during the late Tertiary period when the Andes Mountains were formed (de Cardale-Schrimpff 1978). The sampling sites had varying temperatures (14–20 °C) and pH (5.5–6.5) and exhibited high chloride, sodium, calcium, sulfate and potassium levels (Additional file 1: Table S2). The recovered strains (135) were isolated from these hypersaline environments using several culture media and salinity conditions as described in the methodology section. Sequence analysis of the 16S rRNA (800–1400 bp) gene showed that most strains belonged to the *Actinobacteria*, *Bacteroidetes*, *Firmicutes* and *Proteobacteria* phyla, and only four Archaean strains were obtained, belonging to the genera *Haloferax* in phylum *Euryarchaeota* (Fig. 1).

**Fig. 2:**
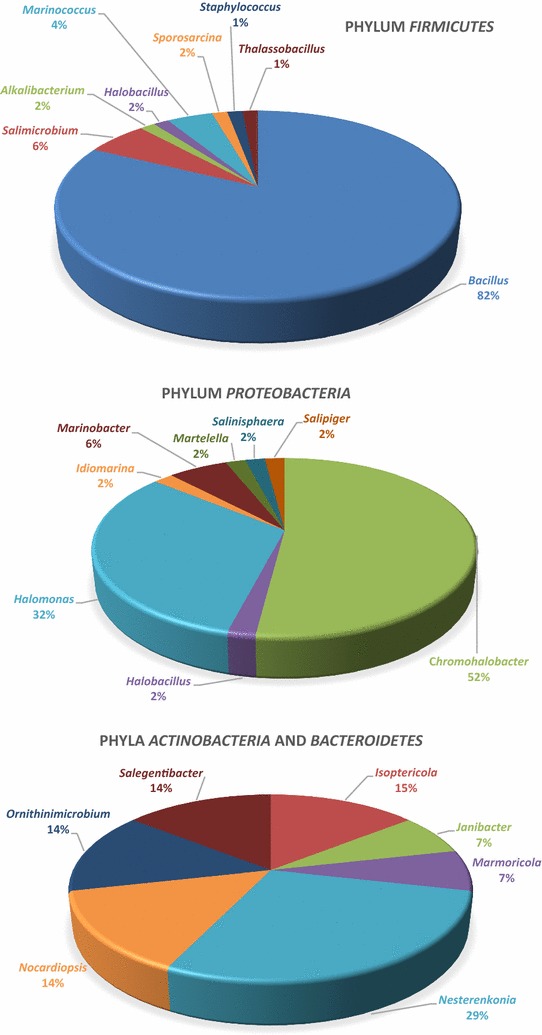
Distribution of the relative abundance of the bacteria genera isolated at Zipaquirá salt mine

The most abundant phylum was *Firmicutes*, represented by the *Bacillaceae*, *Carnobacillaceae*, *Planococcaceae* and *Staphylococcaceae* families. *Bacillus* was the most abundant genus (55 isolates), followed by *Salimicrobium* (4 isolates) and *Marinococcus* (3 isolates). The *Proteobacteria* phylum was represented by *Gammaproteobacteria* and *Alphaproteobacteria* classes, which are commonly found in halophilic environments (Chen et al. 2010). Within *Gammaproteobacteria* we isolated organisms from the *Oceanospirillales* order, in which the *Halomonadaceae* family was the most abundant, with a predominance of *Chromohalobacter* (26 isolates) and *Halomonas* genera (16 isolates). The *Alteromonadales* (*Idiomarinaceae* and *Alteromonadaceae* families) and *Salinisphaerales* orders (*Salinisphaeraceae* family) were also found. Within the *Alphaproteobacteria*, the organisms were distributed between *Rhodobacterales* (*Rhodobacteraceae* family) and *Rhizobiales* (*Aurantimonadaceae* family). *Actinobacteria* were represented by microorganisms of *Janibacter*, *Isoptericola*, *Nesterenkonia*, *Ornithinimicrobium* and *Marmoricola* genera. *Bacteroidetes* was represented by *Salegentibacter* genus (Fig. 2).

### Cytotoxic activity of halophilic isolates

#### Cytotoxic activity of crude extracts

Cytotoxic activity was analyzed for 46 strains randomly selected, looking for including representatives of all genera from the samples collected at Zipaquirá and for 4 strains previously isolated from other saline environments. Ninety-five crude extracts were obtained, 50 of these by partition with chloroform, and 45 by partition with ethyl acetate. Seventeen extracts were not evaluated for their cytotoxic activity because of the yield of the recovered extract (less than 1 mg).

Six *Actinobacteria* strains were evaluated, *Janibacter cremeus* CG 12, *Isoptericola* spp. CG 20 and CG 23, *Ornithinimicrobium kibberense* CG 24, CG 28 and *Nesterenkonia sandarakina* CG 35. Strains CG 23, CG 24, and CG 12 showed high levels of cytotoxic activity (IC_50_ < 60 μg mL^−1^) against the MCF-7 cell line under at least one of the growth conditions evaluated. In contrast, levels of activity against the 4T1 cell line were low (IC_50_ ~ 110 μg mL^−1^) and were only detected in chloroform extracts from the CG 24 and CG 35 strains. Interestingly, the biological activity of strains of the same species was found to vary. For instance, CG 28 and CG 35 strains, which were both identified as *Nesterenkonia sandarakina*, showed variation in activity when grown under the same experimental conditions (Table 1), suggesting that they are not redundant isolates.

**Table 1:** Cytotoxic activities of evaluated strains

Strain ID	Closed type strain (Accession No) (% similarity)	Culture medium	Extraction solvent	Cytotoxic activity of crude extracts IC_50_ (µg mL^−1^)	
				Cellular line	
				4T1	MCF7
Actinobacteria					
CG 23	*Isoptericola halotolerans* (KR476431.1) (98)	TSB 8% (w/v) NaCl	Chloroform	> 250	146.0
		TSB 8% (w/v) NaCl	Ethyl acetate	> 250	53.9
		TSB	Chloroform	–	–
		TSB	Ethyl acetate	–	–
CG 24	*Ornithinimicrobium kibberense* (KM406766.1) (99)	TSB 8% (w/v) NaCl	Chloroform	106.0	> 250
		TSB	Chloroform	–	–
		TSB	Ethyl acetate	> 250	57.2
CG 12	*Janibacter cremeus* (KY775504.1) (94)	TSB 8% (w/v) NaCl	Chloroform	226	34.2
		TSB 8% (w/v) NaCl	Ethyl acetate	> 250	> 250
CG 35	*Nesterenkonia sandarakina* (KF924226) (99)	TSB 8% (w/v) NaCl	Chloroform	117.8	188.6
		TSB 8% (w/v) NaCl	Ethyl acetate	> 250	> 250
CG 28	*Nesterenkonia sandarakina* (KF924226) (98)	TSB 8% (w/v) NaCl	Chloroform	–	–
		TSB 8% (w/v) NaCl	Ethyl acetate	–	–
CG 20	*Isoptericola halotolerans* (KP972642.1) (99)	TSB 8% (w/v) NaCl	Chloroform	–	–
		TSB 8% (w/v) NaCl	Ethyl acetate	–	–
		TSB	Chloroform	–	–
Firmicutes					
CG 6	*Alkalibacterium putridalgicola* (AB681988.1) (99)	Marine broth	Chloroform	134.3	59.9
		TSB	Chloroform	–	–
		TSB	Ethyl acetate	> 250	118.8
CG 3	*Bacillus aquimaris* (KC335217.1) (99)	TSB	Chloroform	–	–
		TSB	Ethyl acetate	> 250	87.3
USBA 882	*Bacillus aquimaris* (NR_025241.1) (96)	Modified marine broth	Ethyl acetate	192.0	NT
USBA 899	*Bacillus hemicentroti* (NR_025264.1) (97)	TSB 3% (w/v) NaCl	Chloroform	–	–
		TSB 3% (w/v) NaCl	Ethyl acetate	–	–
CG 13	*Bacillus aquimaris* (KC335217.1) (92)	TSB 80	Chloroform	–	–
		TSB 80	Ethyl acetate	> 250	200.5
CG 25	*Bacillus hwajinpoensis* (KR045741.1) (97)	TSB 80	Chloroform	–	–
		TSB 80	Ethyl acetate	> 250	56.1
CG 36	*Bacillus aerophilus* (KR010180.1) (99)	TSB	Chloroform	173.2	8.8
CG 42	*Bacillus hwajinpoensis* (KX817927.1) (99)	TSB 3% (w/v) NaCl	Chloroform	–	–
		TSB	Chloroform	–	–
CG 69	*Bacillus aerophilus* (KU236478.1) (99)	TSB	Chloroform	> 250	224.0
CG 11	*Bacillus altitudinis* (NR_118439.1) (99)	TSB	Chloroform	–	–
		TSB	Ethyl acetate	> 250	> 250
CG 7	*Bacillus aquimaris* (NR_113995.1) (95)	TSB	Chloroform	–	–
		TSB	Ethyl acetate	> 250	> 250
CG 69	*Bacillus aerophilus* (KU236478.1) (99)	TSB 3% (w/v) NaCl	Chloroform	–	–
		TSB 3% (w/v) NaCl	Ethyl acetate	–	–
CG 15	*Bacillus licheniformis* (S002290488) (98)	TSB	Chloroform	> 250	136
		TSB	Ethyl acetate	> 250	42.0
		Marine broth	Chloroform	–	–
		Marine broth	Ethyl acetate	> 250	65.6
USBA 866	*Bacillus vietnamensis* (NR_025264.1) (97)	Modified marine broth	Chloroform	> 250	185.7
		Modified marine broth	Ethyl acetate	48.3	65.7
USBA 867	*Bacillus simplex* (NR_109010.1) (98)	Modified marine broth	Ethyl acetate	> 250	134.8
USBA 868	*Bacillus hemicentroti* (NR_114919.1) (99)	Modified marine broth	Ethyl acetate	56.2	76.2
CG 22	*Bacillus subtilis* (CP021499.1) (100)	TSB	Chloroform	*29.8*	*76.5*
		TSB	Ethyl acetate	21.7	75.5
CG 63	*Bacillus amyloliquefaciens* (KY784657.1) (99)	TSB 80	Chloroform	107	76.4
CG 31	*Bacillus weihenstephanensis* (HF678914.2) (99)	TSB 8% (w/v) NaCl	Ethyl acetate	25.4	144.4
CG 57	*Bacillus weihenstephanensis* (KY120752.1) (100)	TSB	Chloroform	–	–
		TSB	Ethyl acetate	–	–
CG 74	*Salimicrobium flavidum* (EU868860.1) (96)	TSB 8% (w/v) NaCl	Chloroform	–	–
		TSB 8% (w/v) NaCl	Ethyl acetate	–	–
CG 86	*Salimicrobium flavidum* (EU868860.1) (96)	TSB 8% (w/v) NaCl	Chloroform	–	–
		TSB 8% (w/v) NaCl	Ethyl acetate	–	–
CG 88	*Salimicrobium flavidum* (EU868860.1) (97)	TSB 8% (w/v) NaCl	Chloroform	–	–
		TSB 8% (w/v) NaCl	Ethyl acetate	–	–
Gammaproteobacteria					
CG 76	*Halomonas alkaliantarctica* (NR_145910.1) (91)	TSB 4% (w/v) NaCl	Ethyl acetate	> 250	96.7
CG X	*Halomonas alkaliantarctica* (NR_145910.1) (91)	TSB 8% (w/v) NaCl	Chloroform	82.3	101.3
CG 60	*Halomonas ventosae* (NR_044519.1) (93)	TSB 8% (w/v) NaCl	Chloroform	–	–
		TSB 8% (w/v) NaCl	Ethyl acetate	> 250	> 250
CG 83	*Halomonas ventosae* (NR_044519.1) (99)	TSB 8% (w/v) NaCl	Chloroform	–	–
		TSB 8% (w/v) NaCl	Ethyl acetate	–	–
USBA 856	*Halomonas ventosae* (NR_042812.1) (99)	Modified marine broth	Chloroform	4.3	217.1
		Modified marine broth	Ethyl acetate	–	–
		TSB	Ethyl acetate	–	–
CG 78	*Halomonas janggokensis* (AB042501.2) (92)	TSB 8% (w/v) NaCl	Chloroform	–	–
		TSB 8% (w/v) NaCl	Ethyl acetate	–	–
CG 66	*Halomonas fontilapidosi* (KT984005.1) (99)	TSB 8% (w/v) NaCl	Chloroform	–	–
		TSB 8% (w/v) NaCl	Ethyl acetate	–	–
USBA 873	*Halomonas taeanensis* (NR_043087.1) (96)	TSB 3% (w/v) NaCl	Chloroform	–	–
		TSB 3% (w/v) NaCl	Ethyl acetate	–	–
CG 50	*Chromohalobacter japonicus* (NR_040965) (97)	TSB 8% (w/v) NaCl	Chloroform	158.6	115
		TSB 8% (w/v) NaCl	Ethyl acetate	–	–
CG 72	*Chromohalobacter japonicus* (NR_040965) (99)	TSB 8% (w/v) NaCl	Chloroform	–	–
		TSB 8% (w/v) NaCl	Ethyl acetate	–	–
CG 55	*Chromohalobacter canadensis* (NR_114545.1) (98)	TSB 8% (w/v) NaCl	Chloroform	–	–
		TSB 8% (w/v) NaCl	Ethyl acetate	69.3	137.6
USBA862	*Chromohalobacter japonicus* (NR_114545.1) (96)	TSB 3% (w/v) NaCl	Chloroform	–	–
		TSB 3% (w/v) NaCl	Ethyl acetate	–	–
USBA 861	*Chromohalobacter japonicus* (NR_114545.1) (95)	TSB 3% (w/v) NaCl	Chloroform	–	–
		TSB 3% (w/v) NaCl	Ethyl acetate	> 250	> 250
USBA 896	*Chromohalobacter canadensis* (NR_114545.1) (96)	TSB 3% (w/v) NaCl	Chloroform	–	–
		TSB 3% (w/v) NaCl	Ethyl acetate	–	–
USBA 344	*Shawanella chilikensis* (BALO01000089) (99)	TSB 3% (w/v) NaCl	Chloroform	5.3	67.9
		TSB 3% (w/v) NaCl	Ethyl acetate	15.3	21.8
CG 65	*Marinobacter persicus* (NR_109110.1) (98)	TSB 8% (w/v) NaCl	Chloroform	–	–
		TSB 8% (w/v) NaCl	Ethyl acetate	–	–
Alphaproteobacteria					
CG 82	*Salipiger nanhaiensis* (NR_134804.1) (98)	TSB	Ethyl acetate	> 250	> 250
USBA 36	*Oceanibaculum indicum* (NR_044547.1) (99)	TSB 5% (w/v) NaCl	Chloroform	173.2	8.8
USBA 85	*Caenispirillum* *bisanense* (NR_04408.1) (98)	TSB	Chloroform	69.6	90.5
		TSB	Ethyl acetate	29.5	74.1
USBA 857	*Martelella mediterránea* (NR_043068.1) (98)	Marine broth	Chloroform	> 250	> 250
USBA 371	*Labrenzia aggregata* (NR_11386.1) (99)	TSB 3% (w/v) NaCl	Chloroform	5.5	4.5

Ten out of the 42 extracts obtained from 23 *Firmicutes* strains belonging to *Alkalibacterium*, *Bacillus* and *Salimicrobium* genera were shown to be cytotoxic against the MCF-7 cell line, with IC_50_ values ranging from 9 to 76 μg mL^−1^. In addition, five extracts showed cytotoxic activity against the 4T1 cell line, with IC_50_ values of under 60 μg mL^−1^. One of the extracts obtained from *Bacillus weihenstephanensis* strain CG 31 presented selective cytotoxicity against the 4T1 cell line, with an IC_50_ of 25.4 μg mL^−1^. No activity was detected in the extracts obtained from the three *Salimicrobium* strains evaluated (CG 74, 86 and 88). As observed with organisms of *Actinobacteria*, the results are strain specific and depend on the growth condition (Table 1).

We evaluated strains of the *Gamma* and *Alphaproteobacteria* classes. Within *Gammaproteobacteria,* 31 extracts obtained from 16 *Halomonas*, *Chromohalobacter, Marinobacter* and *Shewanella* strains were analyzed. *Shewanella chilikensis* strain USBA 344 showed the most cytotoxic activity of any of the extracts obtained in both chloroform and ethyl acetate against both cell lines with an IC_50_ < 15 μg mL^−1^ against the 4T1 cell line, and an IC_50_ < 68 μg mL^−1^ against the MCF-7 cell line. In addition, high levels of cytotoxic activity were detected in the crude extract recovered in chloroform from *Halomonas ventosae* strain USBA 856, which presented an IC_50_ of 4.3 μg mL^−1^. From the six *Chromohalobacter* strains, only the extract obtained from *Chromohalobacter canadensis* CG 55 presented an IC_50_ < 100 μg mL^−1^ against the 4T1 cell line (Table 1).

Extracts of three out of the five strains from the *Alphaproteobacteria* class (*Oceanibaculum, Caenispirillum, Labrenzia*) showed cytotoxic activity (IC_50_ < 100 μg mL^−1^) against one or both cell lines, with the exception of extracts from *Martellela mediterranea* USBA 857 and *Salipiger nanhaiensis* CG 82 that were not active. In particular, two extracts stood out as having strong activity. One of these was the chloroform extract of *Oceanibaculum indicum* strain USBA 36, with an IC_50_ of 9 μg  mL^−1^ against the MCF-7 cell line, and the other was the chloroform extract of *Labrenzia aggregata* strain USBA 371, with IC_50_ values < 5.5 μg mL^−1^ against both cell lines (Table 1).

These results indicate that several strains belonging to diverse bacterial phyla of the halophilic strains recovered in this study are interesting sources of cytotoxic extracts whose cytotoxic compounds are also of interest. The results above allowed selection of CG 12, CG 24, CG 22, CG 31, CG 33, CG 35, CG 50, CG 55, CG 63, CG 76, USBA 344, USBA 85 and USBA 371 strains as the most promising sources of cytotoxic compounds. Differences in the cytotoxicity of one strain at different salt concentrations during culturing on the growth media will be explored in further studies. In addition, isolates identified as taxonomically identical showed different IC_50_ values, suggesting differential production of bioactive metabolites.

#### LC/MS analysis of active extracts and fractions

**Fig. 3:**
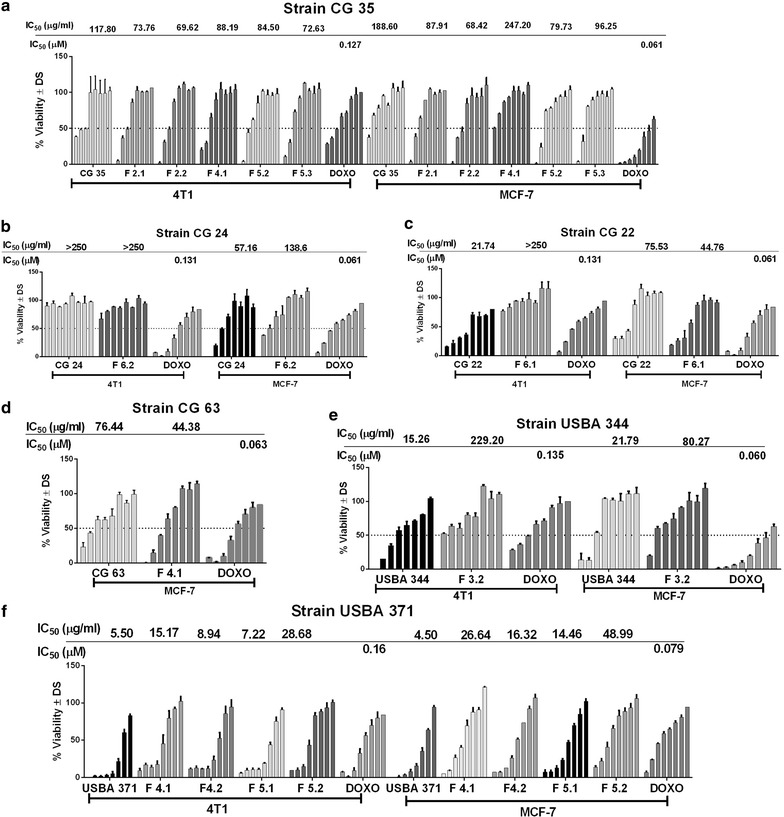
Cytotoxic effects and IC_50_ of crude extracts and fraction from strains CG 35 (**a**), CG 24 (**b**), CG 22 (**c**), CG 63 (**d**), USBA 344 (**e**), and USBA 371 (**f**) on the adherent murine mammary cell carcinoma 4T1 and human mammary adenocarcinoma MCF-7 cell lines. IC_50_ was performed using eight dilutions ranging from 250 to 31.25 μg mL^−1^. *DOXO* Doxorubicin at concentration of 5 μM was used as a positive control

A solid-phase extraction (SPE) fractionation and LC/MS analysis strategy was used to identify compounds present in the extracts of the 13 strains selected. SPE was performed using Diol-SPE cartridges and eluting with solvents of increasing polarity as described in the methods section. Cytotoxicity of the fractions obtained was evaluated, and those active fractions were then analyzed by LC/MS (Table 2, Fig. 3).

**Table 2:** Cytotoxic activities of fractions from the crude extracts

Strain ID	Crude extract/fraction	Extraction solvent	Cytotoxic activity of fractions IC_50_ (µg mL^−1^)	
			Cellular line	
			4T1	MCF7
CG 35	*Crude extract*	*Chloroform*	*117.8*	*188.6*
	F 2.1	Hexane:ethyl acetate 8:2	73.8	87.9
	F 2.2	Hexane:ethyl acetate 8:2	69.6	68.4
	F 4.1	Ethyl acetate	88.2	247.2
	F 5.2	Ethyl acetate:methanol 9:1	84.5	79.7
	F 5.3	Ethyl acetate:methanol 9:1	72.6	96.3
CG 24	*Crude extract*	*Ethyl acetate*	> 250	*57.2*
	F 6.2	Methanol	> 250	138.6
CG 22	*Crude extract*	*Chloroform*	*29.8*	*76.5*
	F 6.1	Methanol	> 250	44.8
CG 63	*Crude extract*	*Chloroform*	*107*	*76.4*
	F 4.1	Ethyl acetate	ND	44.4
CG 50	*Crude extract*	*Chloroform*	158.6	115
	F 6.1	Methanol	31.2	> 250
USBA 344	*Crude extract*	*Ethyl acetate*	*15*	*22*
	F 3.2	Hexane:ethyl acetate 1:1	229.2	80.3
USBA 371	*Crude extract*	*Chloroform*	5.5	4.5
	F 4.1	Ethyl acetate	15.2	26.6
	F 4.2	Ethyl acetate:methanol 9:1	8.9	16.3
	F 5.1	Ethyl acetate:methanol 9:1	7.2	14.5
	F 6.1	Methanol	28.7	49.0

*ND* No data

In some cases, fractionation of the crude extract increased cytotoxic activity against both cell lines. This was the case for the crude extract of *Nesterenkonia sandarakina* CG 35 obtained with chloroform which had an IC_50_ of ≥ 118 μg mL^−1^, while an IC_50_ of less than 88 μg mL^−1^ was detected in the non polar F2.1 and F2.2 (hexane/ethyl acetate 8:2) and medium polarity F5.2 (ethyl acetate/ethanol 9:1) fractions (Fig. 3a).

**Fig. 4:**
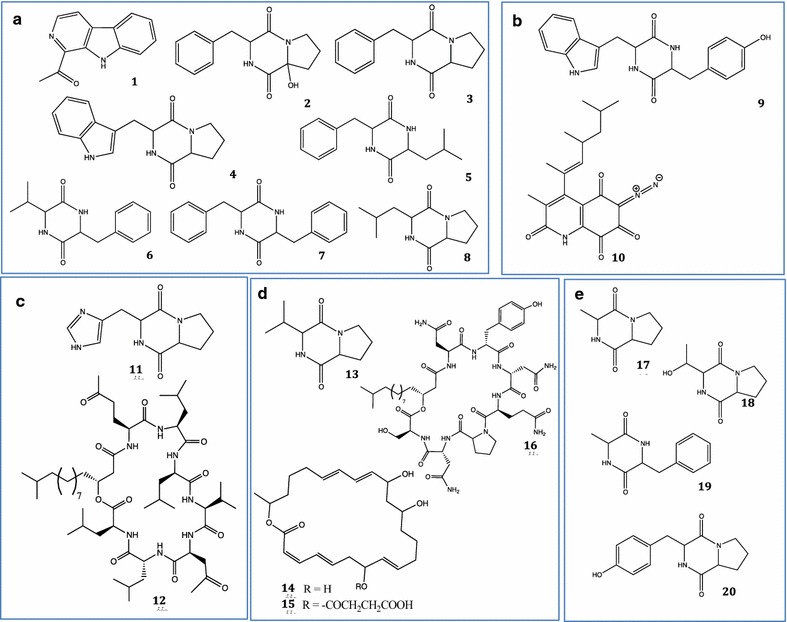
Compounds identified by LC/MS from strains CG 35 (**a**), CG 24 (**b**), CG 22 (**c**), CG 63 (**d**), and CG 50 (**e**). **a** 1-acetyl-β-carboline **1**, *cyclo*(2-OHPro-Phe) **2**, *cyclo*(Pro-Phe) **3**, brevianamide F **4**, *cyclo*(Leu-Phe) **5**, *cyclo*(Val-Phe) **6**, and *cyclo*(Phe-Phe) **7**, *cyclo*(Pro-Leu) **8**; **b** *cyclo*(Trp-Tyr), lagunamycin **10**; **c** *cyclo*(His-Pro) **11**; Surfactin **12**; **d** *cyclo*(Pro-Val) **13**, macrolactin **14**, succinoylmacrolactin **15**, and iturin **16**; **e** *cyclo*(Pro-Ala) **17**, *cyclo*(Pro-Thr) **18**, *cyclo*(Ala-Phe) **19**, *cyclo*(Pro-Tyr) **20**

The LC/MS analysis of the crude extract of *N. sandarakina* CG 35 and dereplication using the Chapman and Hall Dictionary of Natural Products database (Running 1993) and the Fundación Medina data base, allowed identification of 1-acetyl-β-carboline **1**, the diketopiperazines (DKPs) *cyclo*(2-OHPro-Phe) **2**, *cyclo*(Pro-Phe) **3**, brevianamide F **4**, *cyclo*(Leu-Phe)**5**, *cyclo*(Val-Phe) **6**, *cyclo*(Phe-Phe) **7**, *cyclo*(Leu-Pro) **8** (Fig. 4a), and one compound with *m/z* 286.1542 not found in the DNP data base. The LC/MS analysis of the F2.2 fraction, which had the highest cytotoxic activity against both cell lines, also showed the presence of 1-acetyl-β-carboline **1**. The DKPs detected in the crude extract were again obtained from the more polar fractions (F4.1, F5.1 and F5.2) and the presence of two possible new natural compounds was detected in the F2.2 fraction with *m/z* of 211.0869 and 552.2438.

In other cases, fractionation resulted in decreases in cytotoxic activity. The crude extract obtained from *J. cremeus* strain CG 12 had an IC_50_ of 226 μg mL^−1^ against 4T1 and 34 μg mL^−1^ against MCF-7, but upon fractionation activity against the MCF-7 cell line was lost, and activity against the 4T1 cell line was reduced. Only slight activity was observed in the non-polar fractions (F2.2 and F3.2) (data not shown). The decreases in the activity could be related to synergistic effects or analytical procedures that should be optimized in order to increase the recovery of the compounds present in the extracts.

For *O. kibberense* strain CG 24, the IC_50_ decreased after fractionation from 57 to 185 μg mL^−1^ against the MCF-7 cell line, the latter in the F6.2 polar fraction (Table 2, Fig. 3b). LC–MS analysis of the crude extract and the F6.2 fraction revealed the presence of the DKPs *cyclo*(2OHPro-Phe) **2**, *cyclo*(Trp-Tyr) **9** (Fig. 4b), and ions of compounds that did not match the databases here used at *m/z* 235.1189. The clearest difference was the presence of the compound lagunamycin **10** (Fig. 4b) in the crude extract, which was not detected from the active fraction.

Fractionation of extracts from *Bacillus* strains (CG 22, CG 63, CG 31 and CG 33) also gave variable results. While the crude extract of strain CG 22 had cytotoxic activity against both cell lines, only the most polar fraction (F6.1) was active against the MCF-7 cell line (IC_50_ of 45 μg mL^−1^) (Table 2, Fig. 3c). This fraction contained DKPs *cyclo*(His-Pro) **11** (Fig. 4c), *cyclo*(Pro-Phe) **3**, as well as ions for analogs of surfactins such as surfactin A1, 4-l-alaninesurfactin C, surfactin B1 and surfactin A **12** (Fig. 4c), and an unidentified compound with *m/z* = 3416.5966 Da. In the case of strain CG 63, fractionation of the crude extract, which had activity against both cell lines, indicated that only the medium polarity fraction (F4.1) had cytotoxic activity against MCF-7 (IC_50_ value of 44.4 μg mL^−1^) (Table 2, Fig. 3d). In this fraction, *cyclo*(Pro-Phe) **3,** *cyclo*(Pro-Val) **13**, and several stereoisomers of macrolactin **14,** and succinoylmacrolactin **15**, surfactins and iturin **16**, were detected (Fig. 4d). Since cytotoxic activity was lost in fractions from the extracts of strains CG 31 and CG 33 (IC_50_ > 250 μg mL^−1^), these were not analyzed by LC–MS.

Fractionation of extracts from *Chromohalobacter* increased their activity in the case of CG 50 for which the most polar fraction (F6.1) showed an IC_50_ = 31.2 μg mL^−1^ against the 4T1 cell line (Table 2). LC/MS analysis of this fraction again showed the presence of the DKPs *cyclo*(Pro-Ala) **17**, *cyclo*(Pro-Thr) **18**, *cyclo*(Ala-Phe) **19**, *cyclo*(Pro-Tyr) **20** (Fig. 4e) and *cyclo* (Pro-Phe) **3**. In contrast, activity strongly decreases upon fractionation of the extract from strain CG 55, so LC–MS analysis was not performed.

After fractionation of the crude extract of *H. salicampi* CG 76, the cytotoxicity analysis of the recovered fractions showed no activity against the MCF-7 cell line. The initial IC_50_ in the crude extract was 95 μg mL^−1^ against this cell line.

Although *Shewanella chilikensis* strain USBA 344 showed high levels of cytotoxic activity in the crude chloroform (IC_50_ of 35 μg mL^−1^ against 4T1 and 68 μg mL^−1^ against MCF-7) and ethyl acetate extracts (IC_50_ 15 μg mL^−1^ against 4T1 and 22 μg mL^−1^ against MCF-7), after fractionation of these extracts, test showed that cytotoxic activity against the 4T1 cell line had been lost. Conversely, we detected activity from the non-polar fraction F3.2, with an IC_50_ of 80 μg.mL^−1^ against the MCF-7 cell line (Table 2, Fig. 3e). Analysis of the ethyl acetate extract allowed identification of the presence of *cyclo*(Pro-Tyr) **9** and *cyclo*(Pro-His) **11**, along with two unidentified compounds.

Fractionation of *Labrenzia aggregata* USBA 371, which showed the highest levels of cytotoxicity of all the strains evaluated (IC_50_ < 5.5 μg mL^−1^), also resulted in fractions with high levels of activity against both cell lines. The medium polarity F4.1, F4.2 and F5.1 fractions presented IC_50_ values < 16 μg mL^−1^ against the 4T1 cell line and < 27 μg mL^−1^ against MCF-7, whereas the polar fraction F6.1 presented IC_50_ values < 29 μg mL^−1^ against 4T1 and < 49 μg mL^−1^ against MCF-7 (Table 2, Fig. 3f). We detected DKPs *cyclo*(Pro-Phe) **3**, *cyclo*(Leu-Phe) **5**, *cyclo*(Val-Phe) **6,** *cyclo*(Phe-Phe) **7**, *cyclo*(Pro-Leu) **8**, *cyclo*(His-Pro) **11,** *cyclo*(Pro-Val) **13**, *cyclo*(Pro-Ala) **17**, and *cyclo*(Pro-Tyr) **20**.

## Discussion

Halophilic organisms that have evolved and adapted in oligotrophic marine and other saline environments may produce novel molecules with promising biological activity (Zhang 2005). In this study, we therefore analyzed cytotoxic activity against tumor cell lines of halophilic and halotolerant microorganisms isolated from a hypersaline environment. Most of the strains were isolated on halophilic medium and culture media made with saline water from sampling sites and supplemented with several carbon sources. The largest number of isolates were obtained from samples from sampling sites P3 and P5.

Most of these isolates have been previously reported in marine or oceanic environments and other highly saline environments (Claverías et al. 2015; da Silva et al. 2015). Based on the conditions used for recovering these microorganisms, the majority of our isolates are heterotrophic, mesophilic, aerobic or facultative aerobes that are halophilic or halotolerant organisms that can cope with salt concentrations ranging from 0 to 8% NaCl (w/v). Several colonies had yellow, orange, pink and cream pigments, and most were brightened with a creamy texture and were completely round. The exceptions were some *Bacillus* strains whose colonies were opaque with irregular borders. The growth times of most isolates ranged between 24 and 72 h, with some exceptions of up to 10 days for *Actinomycetes*.

A total of 43% of the strains isolated belonged to *Bacillus*. The species of this genus are ubiquitous in various environments and are known for having a large number of biosynthetic gene clusters for the production of secondary metabolites with cytotoxic or antimicrobial activities that promote plant growth or act as biocontrol agents (Ongena and Jacques 2008; Aleti et al. 2015). In this study, we isolated strains of ubiquitous species such as *B. subtilis*, *B. amyloliquefaciens*, *B. pumilus* and *B. licheniformis,* and species typically associated with marine habitats such as *B. aquimaris*, *B. baekryungensis* and *B. hwajinpoensis*. All of these marine strains have been reported in marine sponges with bioactive potential due to the presence of structurally unrelated antimicrobial compounds including polyketides, non-ribosomal synthesized peptides and bacteriocins (Phelan et al. 2012). In our study, we detected polyketides with high levels of cytotoxicity such as macrolactin from extracts of *Bacillus amyloliquefaciens* CG 63, and we detected non-ribosomally synthesized lipopeptides like surfactins and iturins from *Bacillus amyloliquefaciens* CG 63 and from *Bacillus subtilis* CG 22. The cytotoxic activity of macrolactin has been demonstrated previously (Kim et al. 2011; Regmi et al. 2015). Surfactins and iturins are well documented for their antitumor, antifungal, antibacterial, antiviral and insecticidal activities (Vollenbroich et al. 1997; Stein 2005; Kim et al. 2007, 2010; Dey et al. 2015).

Other genera with abundant representatives among our isolates were *Chromohalobacter* and *Halomonas* which have been reported to produce metabolites with antimicrobial and cytotoxic activity. In *Halomonas,* cytotoxic activity with an IC_50_ > 100 μg mL^−1^ has been reported in extracts obtained from *H. ventosae* and *H. salina* (Chen et al. 2010). Promising cytotoxic activity has also been reported in *H. meridiana* and *H. aquamarina* (Sagar et al. 2013). In this study, we detected cytotoxic activity in *Halomonas alkaliantarctica* strain CG 76, and *H. ventosae* strain USBA 856. This latter strain has been studied mainly for its ability to produce exopolysaccharides with physical and chemical properties that confer several bioactive functions (Mata et al. 2006). From the six strains of *Chromohalobacter* evaluated, strains CG 50 and CG 55 showed cytotoxic activity. Sagar et al. (2013) have also reported cytotoxic activity of *Chromohalobacter* strains against the MCF-7 and HeLa (cervical carcinoma) cell lines in lipophilic extracts obtained with chloroform. In this study, we obtained DKPs consisting mainly of proline in the chloroform extract. Similar results were reported by Bitzer et al. (2006) who detected DKPs with a predominance of this amino acid in lipophilic extracts obtained from *Halomonas* spp.

The genera *Nesterenkonia*, *Isoptericola*, *Ornithinimicrobium* and *Janibacter* are included within the rare *Actinobacteria* group and are less frequently isolated than *Streptomyces* (Claverías et al. 2015). They are an important focus of new generation pharmaceutical agents (Mahmoud and Kalendar 2016). The compounds detected in all fractions of these “rare” *actinomycetes* correspond mainly to DKPs. These compounds have also been previously reported in several marine *actinomycetes*, which have emerged as producers of novel DKPs. These include cyclomarazines (Schultz et al. 2008), naseseazines (Raju et al. 2009), nocazines D and E and methoxyneihumicin (Zhang et al. 2013). In addition to DKPs, we isolated 1-acetyl-β-carboline (Shin et al. 2010) from the F2.2 fraction of *Nesterenkonia* sp. strain CG 35. This fraction showed cytotoxic activity against both cell lines (< 70 μg mL^−1^) although, 1-acetyl-β-carboline has not been reported to be a cytotoxic compound. However, it has been shown to have herbicidal, fungicidal and antimicrobial activities, the latter against methicillin-resistant *Staphylococcus aureus* MRSA (Elleuch et al. 2010; Shin et al. 2010).

The strain with the highest levels of cytotoxic activity was *Labrenzia aggregata* USBA 371 which was isolated from the Salado de Consotá salt spring (Díaz-Cárdenas and Baena 2015). Organisms of the *Labrenzia* genus isolated from marine sponges have presented antimicrobial activity against *B. subtilis* and MRSA (Graça et al. 2013). However, no previous reports have identified a cytotoxic effect or have shown that this effect is related to the presence of a mixture of DKPs, as shown in this study. On the other hand, dereplication of the LC/MS data allowed identification of several compounds from the database here used as described below. However, many ions showed no coincidence in the database, which indicates the metabolic potential of the extracts analyzed. This potential will be further evaluated within our group.

Compounds isolated were mainly DKPs, although lipopeptides and polyketides were also isolated. The DKPs are produced by the condensation of two amino acids and are common metabolites of bacteria, fungi and sponges. These compounds can act as signal molecules (Tommonaro et al. 2012) and present antifungal (Nishanth Kumar et al. 2012), antibacterial (Fdhila et al. 2003) and antitumor activities (van der Merwe et al. 2008) due to their chiral and functionalized structure which allows them to bind with high affinity to a great variety of receptors. These compounds exhibit a wide range of biological activities (Martins and Carvalho 2007). Even though we have presented the dereplication of 20 compounds, the data obtained demonstrate enormous chemical diversity to be explored. Studies to this end are currently underway in our laboratories. These strains also represent a starting point for the development of biotechnological processes for obtaining these and other compounds for further studies and applications.

Despite the importance of bioactive compounds for the development of novel therapeutic substances, only a small fraction of microorganisms isolated in pure cultures has been evaluated for production of bioactive molecules. Most studies have focused on a few biological groups other than halophilic microorganisms. Although marine environments have been identified as a major source of drug candidates in clinical trials, and a search for sources of new chemical diversity in the oceans is ongoing, this study demonstrates that terrestrial hypersaline environments have great potential for becoming a major source of bioactive molecules. Our study shows a wide diversity of halophilic and halotolerant bacteria in hypersaline environments, indicative of their great adaptive capacities, and demonstrates that they are versatile producers of bioactive compounds. This work will be the basis for the development of future research on the cytotoxic potential of strains of the genera *Isoptericola*, *Ornithinimicrobium*, *Janibacter*, *Nesterenkonia*, *Alkalibacterium*, *Bacillus, Halomonas*, *Chromohalobacter*, *Shewanella, Martelella, Oceanibaculum, Caenispirillum,* and *Labrenzia*. Compounds identified by LC/MS analysis and dereplication were mainly diketopiperazines. However, some other classes of compounds including polyketides and lipopeptides were also observed, showing a promising chemical diversity to be explored.

## Additional file

**Additional file 1: Table S1.** Cultures conditions for cytotoxic activity screening assays. **Table S2.** Physicochemical characteristics of sampling sites in the salt mine in Zipaquirá.

## Abbreviations

DKPs: diketopiperazines
LC/MS: liquid chromatography–mass spectrometry
ESI: electrospray ionization
TFA-Na: sodium trifluoroacetate
UV-HPLC-HRMS: ultraviolet-high performance liquid chromatography–high resolution mass spectrometry
