# Correction to: Microbial diversity of saline environments: searching for cytotoxic activities

**DOI:** 10.1186/s13568-018-0561-z

**Published:** 2018-03-09

**Authors:** Carolina Díaz-Cárdenas, Angela Cantillo, Laura Yinneth Rojas, Tito Sandoval, Susana Fiorentino, Jorge Robles, Freddy A. Ramos, María Mercedes Zambrano, Sandra Baena

**Affiliations:** 1 Unidad de Saneamiento y Biotecnología Ambiental, Departamento de Biología, Pontificia Universidad Javeriana, POB 56710, Bogotá DC, Colombia; 2 Corporación Corpogen, Carrera 5 # 66A-34, Bogotá DC, Colombia; 3 Grupo de Inmunobiología y Unidad de Investigación en Ciencias Biomédicas, Pontificia Universidad Javeriana, POB 56710, Bogotá DC, Colombia; 4 Grupo de Investigación Fitoquímica, Pontificia Universidad Javeriana, POB 56710, Bogotá DC, Colombia; 5 Departamento de Química, Universidad Nacional de Colombia-Sede Bogotá, Carrera 30 # 45-03, Bogotá DC, Colombia

## Correction to: AMB Expr (2017) 7:223 10.1186/s13568-017-0527-6

The original version of this article (Diaz-Cardenas et al. 2017) unfortunately contained a mistake in Fig. 1. The pie chart of Fig. 1 should explain the distribution of the relative abundance of the Bacteria and Archaea strains isolated at Zipaquirá salt mine: Proteobacteria 39%; Actinobacteria 9%, Bacteroidetes 1%, Archaea 3% and Firmicutes 48% instead of NOMBRE DE CATEGORIA [PORCENTAJE]. The corrected Fig. 1 and caption are given in this erratum.

**Fig. 1:**
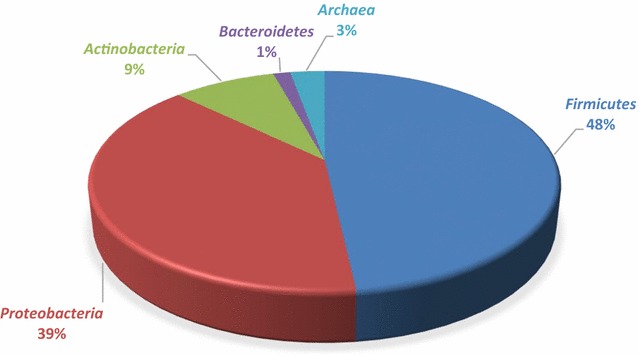
Distribution of the relative abundance of the Bacteria and Archaea strains isolated at Zipaquirá salt mine

The original article has been corrected.
