# Supplementary material for: Microbial diversity of saline environments: searching for cytotoxic activities
Source: AMB Express. 2017 Dec 22;7:223. doi: 10.1186/s13568-017-0527-6 (PMC5741568; doi:10.1186/s13568-017-0527-6)
Supplement: Supplementary file 1 — Additional file 1: Table S1. Cultures conditions for cytotoxic activity screening assays. Table S2. Physicochemical characteristics of sampling sites in the salt mine in Zipaquirá. [file 13568_2017_527_MOESM1_ESM.docx]

**Supplementary material**

**AMB Express**

**Microbial Diversity of Saline Environments: Searching for Cytotoxic Activities**

Carolina Díaz-Cárdenas^1^; Angela Cantillo^2^, Laura Yinneth Rojas^3^, Tito Sandoval^3^, Susana Fiorentino^3^, Jorge Robles^4^, Freddy A Ramos^5^ María Mercedes Zambrano^2^, Sandra Baena^1*^

^1^ Unidad de Saneamiento y Biotecnología Ambiental, Departamento de Biología, Pontificia Universidad Javeriana, POB 56710, Bogotá DC. Colombia.

^2^ Corporación Corpogen. Carrera 5 # 66A-34, Bogotá DC. Colombia.

^3^ Grupo de Inmunobiología y Unidad de Investigación en Ciencias Biomédicas. Pontificia Universidad Javeriana, POB 56710, Bogotá DC. Colombia.

^4^ Grupo de Investigación Fitoquímica, Pontificia Universidad Javeriana, POB 56710, Bogotá DC. Colombia.

^5^ Universidad Nacional de Colombia – Sede Bogotá, Departamento de Química, Carrera 30 # 45-03, Bogotá D.C. Colombia

Corresponding authors:

[baena@javeriana.edu.co](mailto:baena@javeriana.edu.co). Phone +571-3208320 ext 4170. Fax: +571-3208320 ext 4056

**Table S1. Cultures conditions for cytotoxic activity screening assays**

| **Strain ID** | **Closed type strain (Accesion No) (% similarity)** | **Isolation site** | **Culture medium** | **Time (hours)** | **Volume (mL)** | **R.P.M** |
| --- | --- | --- | --- | --- | --- | --- |
|  |  |  |  |  |  |  |
|  |  |  |  |  |  |  |
| ***Actinobacteria*** | | | | | | |
| CG 23 | *Isoptericola halotolerans* (KR476431.1) (98) | P3 | TSB 8% (w/v) NaCl | 120 | 100 | 200 |
|  |  |  | TSB |  |  |  |
| CG 24 | *Ornithinimicrobium kibberense* (KM406766.1) (99) | P3 | TSB 8% (w/v) NaCl | 120 | 100 | 200 |
|  |  |  | TSB |  |  |  |
| CG 12 | *Janibacter cremeus* (KY775504.1) (94) | P3 | TSB 8% (w/v) NaCl | 120 | 100 | 200 |
| CG 35 | *Nesterenkonia sandarakina* (KF924226) (99) | P3 | TSB 8% (w/v) NaCl | 120 | 200 | 200 |
| CG 28 | *Bacillus pumilus (KX185399.1) (99.1)* | P3 | TSB 8% (w/v) NaCl | 48 | 50 | 200 |
| CG 20 | *Isoptericola chiayiensis* (KP972642.1) (81) | P3 | TSB 8% (w/v) NaCl | 120 | 100 | 200 |
|  |  |  | TSB |  |  |  |
| ***Firmicutes*** | | | | | | |
| CG 6 | *Alkalibacterium putridalgicola* (AB681988.1) (99) | P3 | Marine broth | 192 | 60 | 200 |
|  |  |  | TSB |  |  |  |
| CG 3 | *Bacillus aquimaris* (KC335217.1) (96) | P3 | TSB | 48 | 50 | 200 |
| USBA 882 | *Bacillus aquimaris(*NR_025241.1) (96) | P3 | modified marine broth | 48 | 100 | 160 |
| USBA 899 | *Bacillus hemicentroti* (NR_025264.1) (97) | P3 | TSB 3% (w/v) NaCl | 48 | 100 | 160 |
| CG 13 | *Bacillus aquimaris* (KC335217.1) (97) | P3 | TSB 80 | 114 | 60 | 200 |
| CG 25 | *Bacillus hwajinpoensis* (KR045741.1) (97) | P3 | TSB 80 | 120 | 60 | 200 |
| CG 36 | *Bacillus aerophilus* (KR010180.1) (99) | P3 | TSB | 48 | 50 | 200 |
| CG 42 | *Bacillus hwajinpoensis* (KX817927.1) (99) | P4 | TSB 3% (w/v) NaCl | 26 | 50 | 200 |
|  |  |  | TSB | 72 | 60 |  |
| CG 69 | *Bacillus aerophilus* (KU236478.1) (99) | P5 | TSB | 48 | 50 | 200 |
| CG 11 | *Bacillus altitudinis* (NR_118439.1) (99) | P3 | TSB | 48 | 60 | 200 |
| CG 47 | *Bacillus pumilus* | P4 | TSB | 48 | 50 | 200 |
|  |  |  |  |  |  |  |
| CG 7 | *Bacillus aquimaris* (NR_113995.1) (95) | P3 | TSB | 72 | 60 | 200 |
| CG 69 | *Bacillus aerophilus* (KU236478.1) (99) | P5 | TSB 3% (w/v) NaCl | 26 | 50 | 200 |
| CG 15 | *Bacillus licheniformis*  (S002290488) (98) | P5 | TSB | 48 | 50 | 200 |
|  |  |  | Marine broth |  |  |  |
| USBA 866 | *Bacillus vietnamensis* (NR_025264.1)(97) | P5 | Modified marine broth | 72 | 50 | 180 |
|  |  |  |  |  |  |  |
| USBA 867 | *Bacillus simplex* (NR_109010.1) (98) | P3 | Modified marine broth | 48 | 50 | 180 |
| USBA 868 | *Bacillus hemicentroti* (NR_114919.1) (99) | P3 | Modified marine broth | 48 | 50 | 180 |
| CG 22 | *Bacillus subtilis* (CP021499.1) (100) | P3 | TSB | 48 | 100 | 200 |
| CG 63 | *Bacillus amyloliquefaciens* (KY784657.1) (99) | P5 | TSB 80 | 48 | 1000 | 200 |
| CG 31 | *Bacillus weihenstephanensis* (HF678914.2) (99) | P3 | TSB 8% (w/v) NaCl | 48 | 100 | 200 |
| CG 57 | *Bacillus weihenstephanensis* (KY120752.1) (100) | P5 | TSB | 48 | 50 | 200 |
| CG 33 | *Bacillus sp.* | P3 | TSB | 48 | 100 | 200 |
| CG 74 | *Salimicrobium flavidum* (EU868860.1) (96) | P5 | TSB 8% (w/v) NaCl | 48 | 60 | 200 |
| CG 86 | *Salimicrobium flavidum* (EU868860.1) (96) | P5 | TSB 8% (w/v) NaCl | 192 | 60 | 200 |
| CG 88 | *Salimicrobium flavidum* (EU868860.1) (97) | P5 | TSB 8% (w/v) NaCl | 192 | 60 | 200 |
| ***Gammaproteobacteria*** | | | | | | |
| CG 76 | *Halomonas alkaliantarctica* (NR_145910.1) (91) | P5 | TSB 4% (w/v) NaCl | 65 | 100 | 200 |
| CG X | *Halomonas alkaliantarctica* (NR_145910.1) (91) | P5 | TSB 8% (w/v) NaCl | 65 | 100 | 200 |
| CG 60 | *Halomonas ventosae* (NR_044519.1) (93) | P5 | TSB 8% (w/v) NaCl | 48 | 60 | 200 |
| CG 83 | *Halomonas ventosae* (NR_044519.1) (99) | P5 | TSB 8% (w/v) NaCl | 48 | 60 | 200 |
| USBA 856 | *Halomonas ventosae* (NR_042812.1) (99) | P3 | Modified marine broth | 48 | 100 | 160 |
| CG 70 | *Halomonas* sp. | P5 | TSB | 72 | 70 | 200 |
| CG 78 | *Halomonas janggokensis* (92) (AB042501.2) | P5 | TSB 8% (w/v) NaCl | 72 | 60 | 200 |
| CG 66 | *Halomonas fontilapidosi* (99) (KT984005.1) | P5 | TSB 8% (w/v) NaCl | 72 | 60 | 200 |
| USBA 873 | *Halomonas taeanensis* (NR_043087.1) (96) | P5 | TSB 3% (w/v) NaCl | 48 | 100 | 160 |
| CG 50 | *Chromohalobacter japonicus*  (NR_040965) (97) | P5 | TSB 8% (w/v) NaCl | 65 | 100 | 200 |
| CG 72 | *Chromohalobacter japonicus* (NR_040965) (99) | P5 | TSB 8% (w/v) NaCl | 48 | 60 | 200 |
| CG 55 | *Chromohalobacter canadensis*  (NR_114545.1) (98) | P5 | TSB 8% (w/v) NaCl | 65 | 100 | 200 |
| USBA862 | *Chromohalobacter japonicus* (NR_114545.1) (96) | P5 | TSB 3% (w/v) NaCl | 48 | 100 | 160 |
| USBA 861 | *Chromohalobacter japonicus* (NR_114545.1) (95) | P5 | TSB 3% (w/v) NaCl | 48 | 100 | 160 |
| USBA 896 | *Chromohalobacter canadensis* (NR_114545.1) (96) | P3 | TSB 3% (w/v) NaCl | 48 | 100 | 160 |
| USBA 344 | *Shewanella chilikensis* (BALO01000089) (99) | Salpa saline spring | TSB 3% (w/v) NaCl | 48 | 100 | 160 |
| CG 65 | *Marinobacter persicus* (NR_109110.1) (98) | P5 | TSB 8% (w/v) NaCl | 48 | 60 | 200 |
| ***Alphaproteobacteria*** | | | | | | |
| CG 82 | *Salipiger nanhaiensis*  (NR_134804.1) (98) | P5 | TSB | 48 | 60 | 200 |
| USBA 36 | *Oceanibaculum indicum* (NR_044547.1) (99) | Consotá saline spring | TSB 5% (w/v) NaCl | 120 | 100 | 160 |
| USBA 85 | *Caenispirillum bisanense* (NR_04408.1) (98) | Consotá saline spring | TSB | 48 | 100 | 160 |
| USBA 857 | *Martelella mangrovi* (NR_043068.1) (98) | P3 | Marine broth | 48 | 100 | 160 |
| USBA 371 | *Labrenzia aggregata* (NR_11386.1) (96) | La Cristalina saline spring | TSB 3% (w/v) NaCl | 48 | 100 | 160 |

**R.P.M: revolutions per minute**

**Table S2. Physicochemical characteristics of sampling sites in the salt mine in Zipaquirá**

| **Sample ID** | **Temperature**  **C°** | **pH** | **Salinity**  **Conductivity mS.cm^-1^** | **Concentrations mg. L^-1^** | | | | | | | | | | | | |
| --- | --- | --- | --- | --- | --- | --- | --- | --- | --- | --- | --- | --- | --- | --- | --- | --- |
|  |  |  |  | **Ca^2+^** | **Total organic carbon** | **Cl^-^** | **Total**  **PO4^3-^** | **Total**  **Fe** | **Mg^2+^** | **Mn^2+^** | **N-NO_3_** | **N-NH_4_** | **K^+^** | **Na^+^** | **SO_4_^2-^** | **SO_3_^2-^** |
| **P1** | 14.4 | 5.6 | 336 | 4990 | <0,25 | 465000 | <0,10 | 5,75 | 710 | 3,1 | 0,84 | 257,6 | 2635 | 29800 | 2403,4 | <3,0 |
| **P2** | 17 | 6.3 | 340 | 5080 | 2,01 | 710000 | <0,1 | 2,04 | 1050 | 4,8 | 2,48 | 375,2 | 8533 | 30400 | 769,9 | <3 |
| **P3** | 17.3 | 6.6 | 328 | 2270 | <0,25 | 100000 | <0,1 | 1,82 | 46 | 0,56 | 0,39 | 46,8 | 122 | 28600 | 2461,8 | 11 |
| **P4** | 19.2 | 7.1 | 330 | 2400 | <0,25 | 725000 | <0,1 | 2,35 | 44 | 0,65 | 0,37 | 36,4 | 191,4 | 27800 | 1971,6 | <3,0 |
| **P5** | 17.9 | 6.0 | 280 | 3580 | <0,25 | 565000 | <0,1 | 2,16 | 370 | 1,38 | 0,93 | 73,9 | 1455 | 26000 | 1687,5 | <3,0 |
| **P6** | 16.4 | 6.24 | 304 | 2810 | <0,25 | 635000 | <0,1 | 1,99 | 230 | 0,91 | 0,53 | 48,2 | 724 | 26800 | 1858 | <3 |
